# Evaluation of the value of Preoperative Sialic Acid Levels in Diagnosis and Localization of Urothelial Tumors

**DOI:** 10.7150/jca.45648

**Published:** 2021-06-16

**Authors:** Dongshan Chen, Dawei Li, Zhanwu Cui, Cong Zhang, Zhao Zhang, Lei Yan

**Affiliations:** 1 Department of Urology, Qilu Hospital of Shandong University, Wenhuaxi Road 107#, Jinan, 250012, P.R. China.; 2 Department of Urology, Second Traditional Chinese Medicine Hospital of Dezhou City, Zhongxing Road 245#, Dezhou , 253500, P.R. China.

**Keywords:** sialic acid, urothelial tumor, diagnosis, localization

## Abstract

**Objective:** To explore SA levels in the serum of urothelial tumor patients and their correlation with clinical pathological features and localization.

**Materials and Methods:** Our research retrospectively collected data from 591 patients with urothelial tumors between July 2014 and April 2018. The SA levels in the serum of urothelial tumor patients and their correlation with clinical pathological features and localization were investigated. Univariate and multivariate logistic regression analyses were further performed to identify independent associations.

**Results:** The levels of SA were significantly associated with the malignant degree (tumor grade and infiltration) of bladder cancer and tumor localization (all *p* < 0.05). The multivariate logistic regression model showed that SA levels were independently associated with the presence of high-grade urothelial carcinoma (BUC: HR = 1.941, UTUC: HR = 3.820, all *p* <0.05) and upper urinary tract urothelial carcinoma (HR = 2.047, *p* < 0.05). Finally, we validated the diagnosis and localization value of SA in an independent cohort from another institutions.

**Conclusions:** Elevated serum SA levels are an independent predictor of high-grade urothelial carcinoma and upper urinary tract urothelial carcinoma, indicating that SA levels may be a potential biomarker for the diagnosis, prognosis and localization of urothelial tumors.

## Introduction

Urothelial carcinoma (UC) is the ninth most common cancer and is the thirteenth most common cause of death due to cancer, accounting for approximately 430,000 new cases and 165,000 mortalities globally 1, 2. In China, there are 80,500 incident cases and 32,900 mortalities caused by UC each year 3. UC is the most frequent type of cancer of the bladder (BUC) and upper urinary tract 4, while the morbidity and mortality of UC have gradually increased over recent years. Therefore, early detection and diagnose have an important significance to the prognosis and quality of life of patients with UC. Cytological experiments and biopsies under an ureteroscope and a bladder scope are currently the two main means of UC early detection and diagnosis 5. However, use of the ureteroscope and bladder scope not only is invasive but also has a high rate of missed diagnosis, especially for UC *in situ* and upper tract urothelial carcinoma (UTUC) 5, 6. Therefore, the search for a specific, highly sensitive marker, that can predict the biological behavior of UC, as an index for predicting tumor progression and metastasis, in order to guide clinical diagnosis and therapy is particularly important.

Sialic acid (SA) is a monosaccharide with a nine-carbon backbone that occupies the terminal position on macromolecules and cell membranes 7. SA is involved in autoimmune diseases, microbial invasion, virulence pathogenesis and tumor growth 8. Malignant cells often have an increased concentration of SA on the surface and secrete SA to increase the concentration in blood 9. In previous studies, it was found that the lipid-bound subfraction of SA concentration was of limited value for detecting early stages of genitourinary malignancies 10. However, small sample sizes, no further analyses of multiple factors, no assessment of localization values and no validated cut-off values existed in these studies. Therefore, the present study aimed to assess SA levels in patients with UC to determine the pathogenesis and development of UC.

## Materials and methods

### Patients

From July 2014 to April 2018, the clinical data of 591 patients with urothelial carcinoma who underwent surgical treatment in the Department of Urology, Qilu Hospital of Shandong University, were retrospectively analyzed. Among 591 UC patients, 406 patients were diagnosed with bladder tumors, 99 with ureteral tumors and 86 with renal pelvic tumors. Additionally, a total of 322 consecutive UC patients (including 185 BUC patients, 77 ureteral carcinoma patients and 60 renal pelvis carcinoma) treated at Shandong Provincial Hospital between July 2017 to Mar 2021 were used for validation set.

The inclusion criteria for the patients enrolled in the study were as follows:

- Patients with a pathological diagnosis of urothelial tumors, including bladder tumors, ureteral tumors and renal pelvic tumors, and patients who underwent surgical treatment at Qilu Hospital of Shandong University;
- Patients with complete clinical and pathological data, such as preoperative blood parameters, postoperative pathological results and other basic information;
- Patients who did not undergo any adjuvant treatments, such as radiotherapy or chemotherapy, before surgery.

The exclusion criteria for the patients enrolled in the study were as follows:

- The coexistence of any other malignant tumor;
- A pathological diagnosis of nonurothelial tumors;
- A history of bladder urothelial tumors and upper tract urothelial tumors;
- A history of cerebral infarction and cerebral hemorrhage within the last 1 month or myocardial infarction within the latest 6 months;
- The administration of procoagulant or anticoagulant drugs within the past 2 weeks;
- The presence of obvious infection or inflammation.

### Data collection

Clinical data including patient age at the time of diagnosis, sex, smoking history, routine blood examination results (white blood cell count, platelet count, plasma fibrinogen level, etc.), and tumor characteristics were obtained from the electronic patient records at our institution.

### SA measurement

Before any clinical treatment, 5 mL venous blood samples from patients after 8 hours of fasting were collected for the evaluation of SA levels. Blood samples are stored in tubes containing clotting activators and gels. The Roche Cobas 8000 automatic analyzer was used for the determination of SA in serum. SA levels of 45.6-75.4 mg/dL were defined as normal.

### Statistical analysis

The Kolmogorov-Smirnov test was adopted to assess whether the level of SA conformed to a normal distribution, and the values were presented as the mean ± standard deviation (SD). Student's t-test and one-way ANOVA were used for normally distributed data; if the data distribution was not normal, the Mann-Whitney U-test and Kruskal-Wallis H-test were employed for the nonparametric analysis. Data were analyzed and processed using the Statistical Package for Social Sciences version 22.0 (SPSS Inc., Chicago, IL, USA). *P* values < 0.05 in two-tailed tests were considered statistically significant.

## Results

### Clinicopathological characteristics of patients

**Figure 1:**
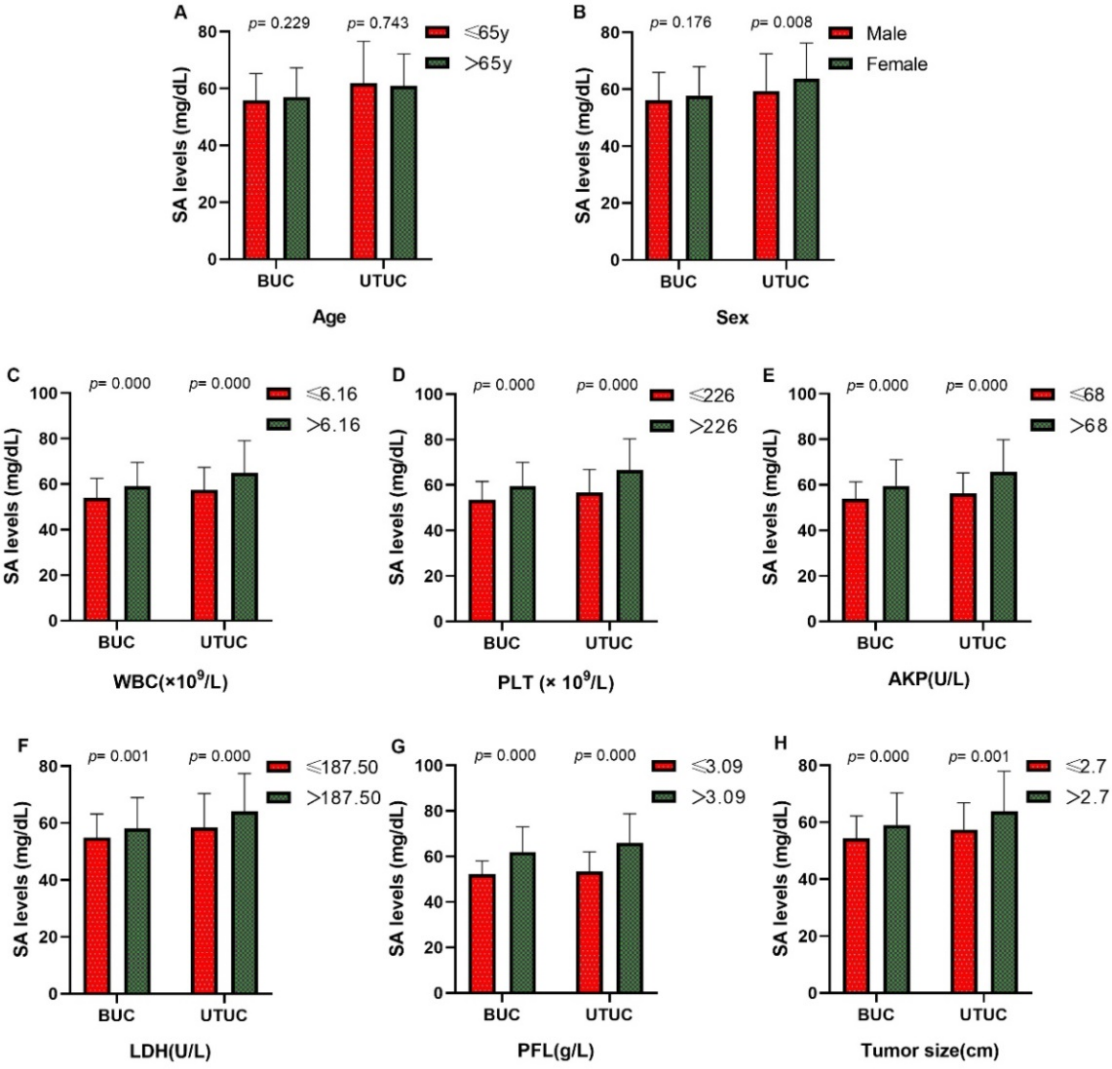
The comparisons of serum SA levels in various groups of patients with urothelial tumor. (A) Age; (B) sex; (C) WBC; (D) PLT; (E) AKP;(F) LDH; (G) PFL; (H) tumor size.

**Figure 2:**
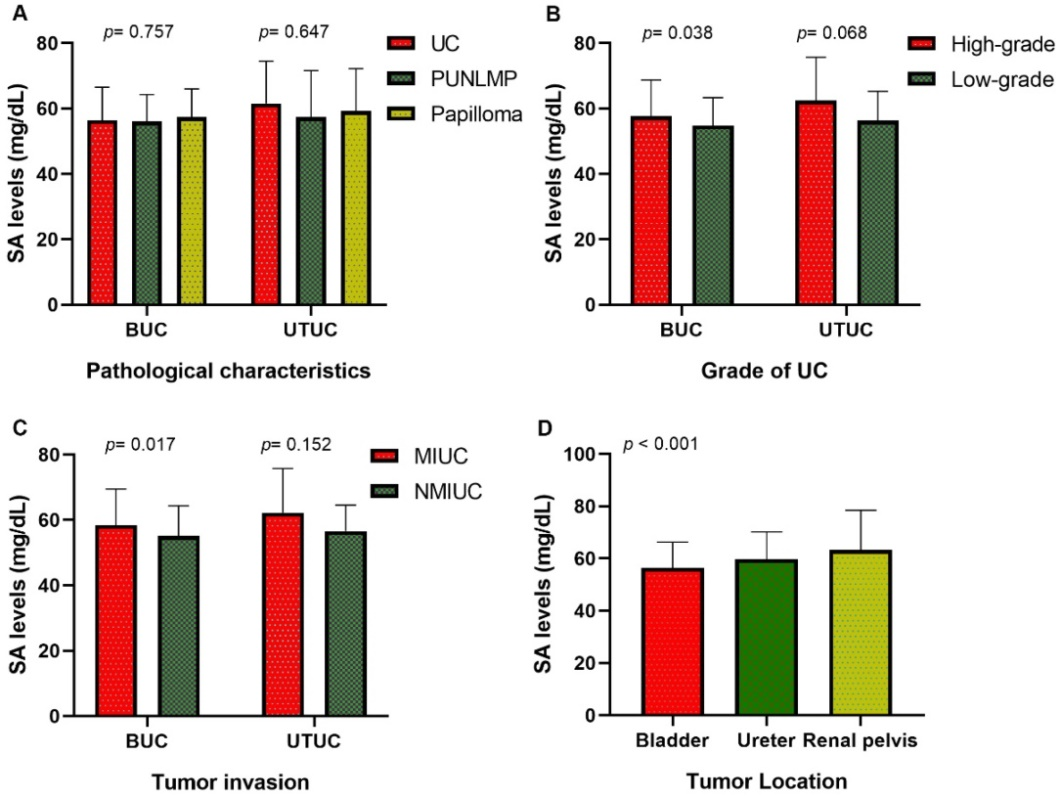
Comparison of SA levels (A) among UC, PUNLMP and papilloma patients; (B) between high-grade and low-grade UC patients; (C) between MIUC and NMIUC patients; (D) among bladder urothelial carcinoma, ureteral tumor and renal pelvis tumor patients.

Our research collected data from 591 patients with urothelial tumors; 448 (75.80 %) were male, and 143 (24.20%) were female. The median age was 65 years, ranging from 17 to 94 years. The mean serum SA level of all patients was 56.41 ± 9.85 mg/dL and 61.40 ± 12.93 mg/dL for BUC and UTUC respectively. There was a significant correlation between serum SA levels and sex, WBC, PLT, AKP, LDH, PFL, and tumor size (all *p <* 0.05, Fig. 1). Most importantly, the serum levels of SA in advanced-stage patients were significantly higher than those in early-stage patients (high-grade UC vs. low-grade UC: 57.69 ± 11.06 vs. 54.84 ± 8.52 mg/dL [BUC, *p* < 0.05], 62.40 ± 13.32 vs. 56.32 ± 8.98 mg/dL [UTUC, *p* = 0.068], Fig. 2B; MIUC vs. NMIUC: 58.40 ± 11.11 vs. 55.07 ± 9.31 [BUC, *p* < 0.05], 62.21 ± 13.54 vs. 56.61 ± 7.94 [UTUC, *p* = 0.152], Fig. 2C). According to the previously collected data, 517 patients (87.48%) developed urothelial carcinoma, 60 patients (10.15%) developed PUNLMP, and 14 patients (2.37%) developed papilloma. However, there was no statistically significant difference in SA levels among UC, PUNLMP and papilloma cases (BUC: *p* = 0.757, UTUC: *p* = 0.647, Fig. 2A). The clinical characteristics of the enrolled patients are shown in Table 1.

### The role of SA level in the localization diagnosis of UC

Of all patients, 406 (68.70%) had bladder tumors, 99 (16.75%) had ureteral tumors, and 86 (14.55%) had renal pelvic tumors. Our study found that the serum SA level in the renal pelvic tumor and ureteral tumor groups was significantly higher than that in patients with bladder tumors (*p* < 0.05, Fig. 2D and Table 2), indicating that the serum SA level may have valuable applications in the localization diagnosis of urothelial tumors.

### Correlation analyses

**Figure 3:**
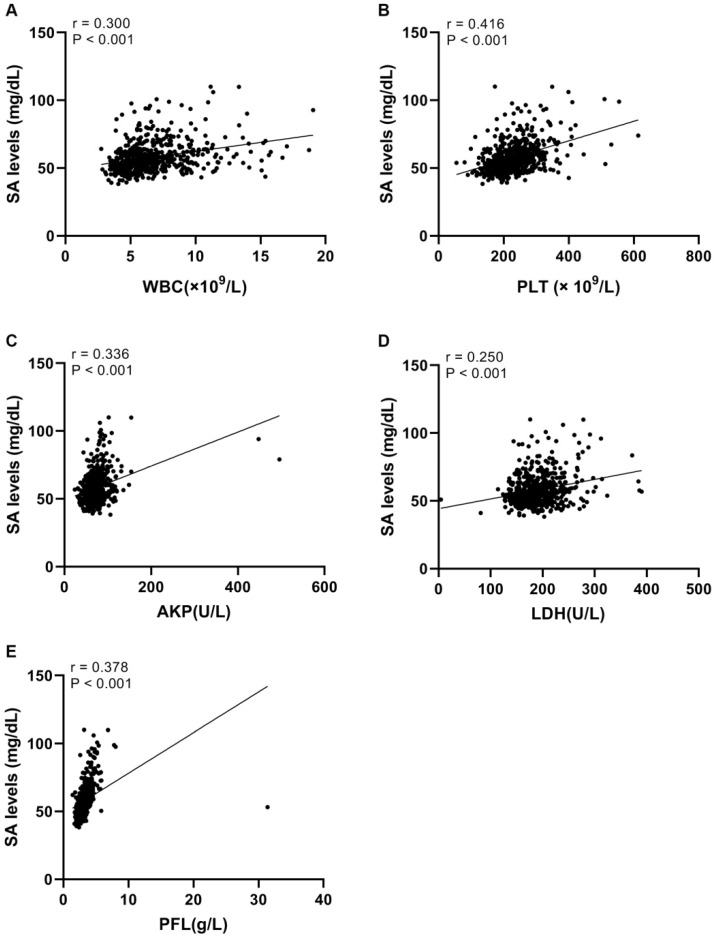
Linear correlations between SA levels and WBC, PLT, AKP, LDH, and PFL in urothelial tumor patients. (A) Linear correlation between SA levels and WBC. (B) Linear correlation between SA levels and PLT. (C) Linear correlation between SA levels and AKP. (D) Linear correlation between SA levels and LDH. (E) Linear correlation between SA levels and PFL.

Linear correlation analyses were conducted to further explore correlations between SA levels and WBC, PLT, LDH, AKP, and PFL in UC patients. We found that there was a significant positive relationship between serum SA levels and WBC (r = 0.300, *p* <0.05, Fig. 3A), PLT (r = 0.416, *p* <0.05, Fig. 3B), AKP (r = 0.336, *p* <0.05, Fig. 3C), LDH (r = 0.250, *p* <0.05, Fig. 3D), and PFL (r = 0.378, *p* <0.05, Fig. 3E).

### The value of serum SA level in accurate location and qualitative diagnosis for UC patients

**Figure 4:**
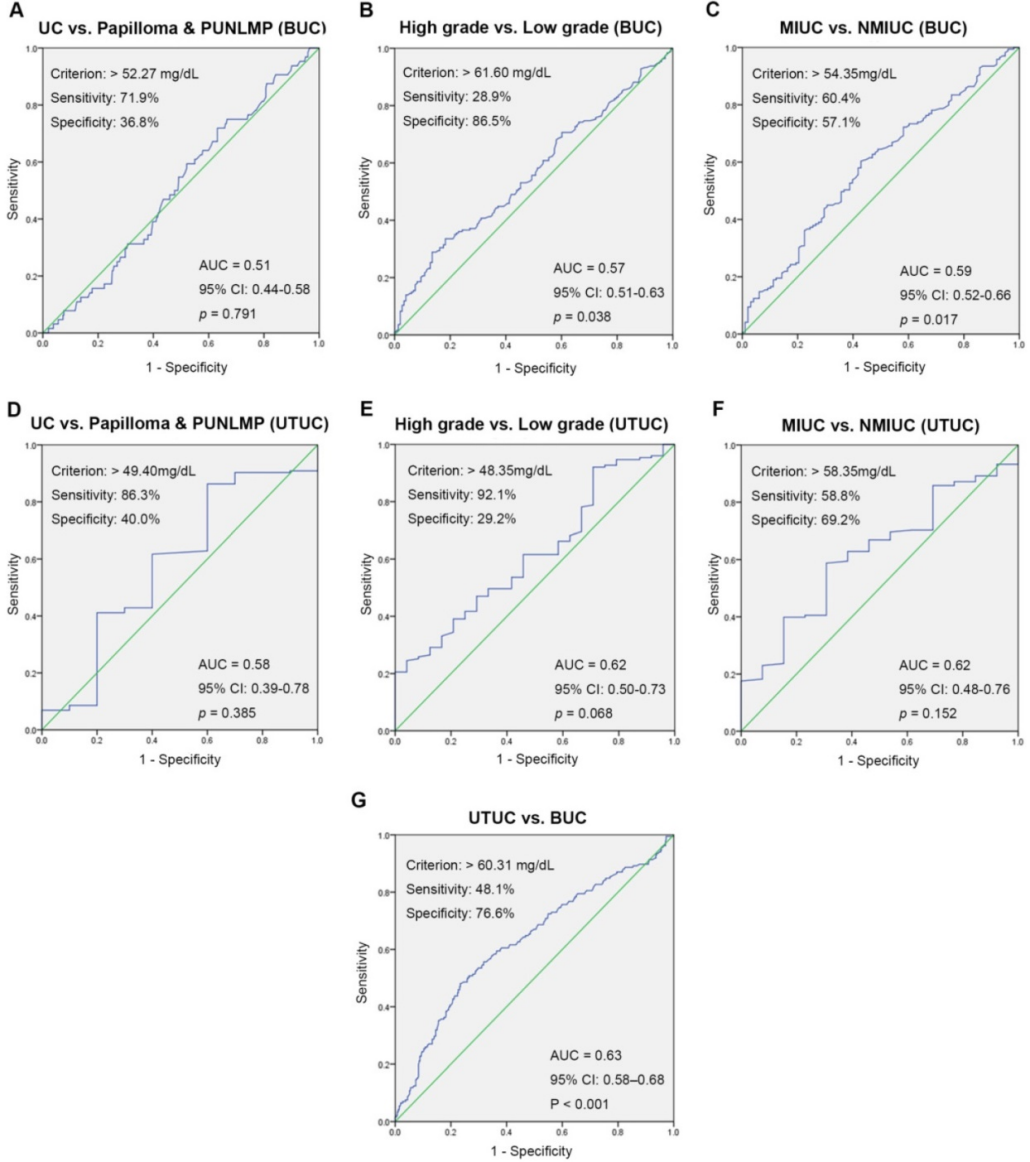
ROC curves for determination of the cut-off value of SA levels regarding the prediction of UC, high-grade UC, MIUC and UTUC.

ROC curve analysis for preoperative SA levels was used to evaluate the value of localization and qualitative diagnoses. The optimal threshold values for preoperative SA levels were > 52.27 mg/dL, > 49.40 mg/dL (UC vs. papilloma and PUNLMP, BUC and UTUC), > 61.60 mg/dL, > 48.35 mg/dL (high-grade UC vs. low-grade UC, BUC and UTUC), > 54.35 mg/dL, > 58.35 mg/dL (MIUC vs. NMIUC, BUC and UTUC), and 60.31 mg/dL (UTUC vs. BUC). Remarkably, there was no clinical value of SA levels in differential diagnosis of UC and papilloma and PUNLMP (BUC: *p* = 0.791; UTUC: *p* = 0.385). Fig. 4 also showed the areas under the ROC curve (AUC), sensitivity and specificity for accuracy of each threshold values.

Univariable and multivariable logistic regression analyses were conducted to further explore the clinical application value of serum SA levels for predicting the prognosis and orientation of urothelial tumors. The univariate analysis showed that there was a significant correlation between elevated preoperative SA levels and tumor orientation (HR = 3.305, p < 0.05) and poor clinical pathology diagnoses (UC: HR = 1.491 [BUC, *p* = 0.183], HR = 4.194 [UTUC, *p* < 0.05]; high-grade: HR = 2.597 [BUC, *p* < 0.05], HR = 4.770 [UTUC, *p* < 0.05]; MIUC: HR = 2.030 [BUC, *p* < 0.05], HR = 3.209 [UTUC, *p* = 0.062]; Table 3). Furthermore, tumor size was a significant predictor of tumor orientation (HR = 2.063, *p* < 0.05), malignant degree and prognosis (UC: HR = 3.744 [BUC, *p* < 0.05], HR = 1.692 [UTUC, *p* = 0.419]; high-grade: HR = 3.137 [BUC, *p* < 0.05], HR = 0.520 [UTUC, *p* = 0.191]; MIUC: HR = 4.226 [BUC, *p* < 0.05], HR = 3.917 [UTUC, *p* = 0.029]; [Table 3]). The univariate analysis results of other relevant variables were displayed in Table 3. Subsequently, sex, LDH, PFL, tumor size and SA level were incorporated into multivariate analysis. We found that SA level > 61.60 mg/dL, SA level > 48.35 mg/dL and SA level > 60.31 mg/dL were independent risk factors for high-grade BUC (HR = 1.941, *p* < 0.05; Table 4), high-grade UTUC (HR = 3.820, *p* < 0.05; Table 4), and UTUC (HR = 2.047, *p* < 0.05; Table 4), respectively. In addition, tumor size was independent risk factors for poor clinicopathology outcomes in BUC patients (UC: HR = 3.924, high grade: HR = 2.802, MIUC: HR = 3.985; all *p* < 0.05, Table 4), and was a predictor for the tumor localization (UTUC: HR = 1.660, *p* < 0.05, Table 4).

### Validation in an independent cohort

**Figure 5:**
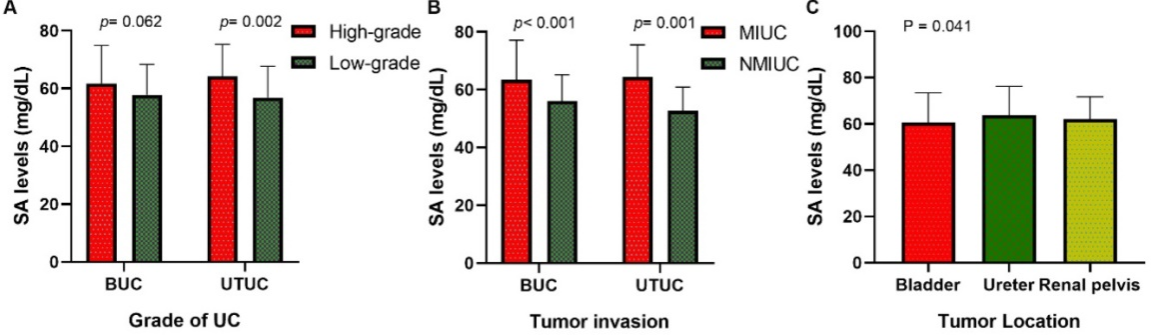
Comparison of SA levels of validation set (A) between high-grade and low-grade UC patients; (B) between MIUC and NMIUC patients; (C) among bladder urothelial carcinoma, ureteral tumor and renal pelvis tumor patients.

To find out whether the effect of SA was applicable for other UC cases, we reviewed the medical records of 322 UC patients from another institutions as an independent cohort. Our study validates the serum levels of SA in advanced-stage patients were significantly higher than those in early-stage patients (high-grade UC vs. low-grade UC: 61.70 ± 13.27 vs. 57.83 ± 10.56 mg/dL [BUC, *p* = 0.062], 64.14 ± 11.13 vs. 56.91 ± 10.77 mg/dL [UTUC, *p* < 0.05], Fig. 5A; MIUC vs. NMIUC: 63.40 ± 13.67 vs. 56.00 ± 9.18 [BUC, *p* < 0.05], 64.53 ± 10.97 vs. 52.72 ± 8.20 [UTUC, *p* < 0.05], Fig. 5B). In addition, serum SA level was obviously elevated in UTUC patients compared to BUC patients (*p* < 0.05, Fig. 5C). The detailed clinicopathological data was presented in Table 5 and Table 6.

## Discussion

Urothelial carcinoma can be divided mainly into UTUC and BUC, and has a notably high rate of clinical variability, recurrence, progression, and cancer-specific mortality 11, 12. UTUC has symptoms and signs similar to those of BUC 13, whereas UTUC is markedly more aggressive and seems to have a higher burden of nodal metastases at diagnosis than BUC 14, 15. Our current diagnostic and detection methods for UC are invasive, radioactive and expensive procedures 16, such as CT, MRI and pathology. Therefore, there is an urgent need to find an effective, simple and noninvasive method for the diagnosis, orientation and guidance of clinical treatments for UC.

Sialic acids, a family of monosaccharides with negative charges, are typically located at the terminal positions of cell surface glycoproteins and glycolipids 17. Due to their special physical and chemical properties, such as charge and size, SAs are involved in a variety of biologically important processes and influence both the degree of severity and the progression of disease in a range of illnesses. Moreover, the combinatorial diversity enables SAs to influence the structure and function of glycoproteins and lipids as well as to regulate cell-cell and cell-extracellular matrix interactions 18. For example, sialic acids can mediate pathogen infection, immunogenicity, cell adhesion and migration, vascularization and differentiation and can serve as ligands of sialic acid-binding proteins, such as factor H, selectins and Siglecs 19-21.

There are quantitative and qualitative changes in the expression of cellular surface molecules in the canceration course, which is crucial for the unlimited proliferation and malignant behavior of neoplastic cells 22. Glycoconjugates including glycoproteins and glycolipids, are ubiquitous, essential components of cell membranes and therefore participate in malignant transformation and tumor progression 23. The aberrant expression of SA facilitates cancer cell migration and metastasis formation and is an important factor in tumor immunological escape and the immunosuppressive microenvironment 24-26. Strikingly, these glycoconjugates are released into the circulation through increased turnover, secretion and/or shedding from malignant cells, which results in increased SA levels in the blood 9, 27. Previous studies have confirmed that there is marked clinical significance for measuring serum SA in the diagnosis and treatment evaluation of several solid tumors, including colorectal cancer 28, osteosarcoma 29, oral squamous cell carcinoma 27, pancreatic cancer 30, melanoma 26, and prostatic cancer 31.

In the present study, we examined preoperative SA levels and other clinical features of 591 patients with newly diagnosed urothelial tumors. We found that the extent of malignancy was significantly correlated with serum SA levels. Meanwhile, we noticed that the SA level in patients with renal pelvis carcinoma and ureteral tumors was higher than that in patients with bladder tumors. To further understand the association between SA levels and other clinical features, linear correlation analyses were performed and showed that serum indicators such as WBC, PLT, LDH, AKP, and PFL were linearly correlated with SA levels. Furthermore, ROC curves further confirmed the practical application value of SA levels in the diagnosis and localization of urothelial tumors. The optimal cut-off values with the maximum Youden index were > 61.60 mg/dL (BUC: high-grade vs. low-grade), 54.35 mg/dL (BUC: MIUC vs. NMIUC), and 60.31 mg/dL (UTUC vs. BUC). Logical regression analysis showed that the SA level was a powerful independent predictor of high-grade UC and UTUC, which supported the potential utility of SA levels in clinical practice as a noninvasive biomarker for the diagnosis and localization of urothelial tumors. Finally, we validated the diagnosis and localization value of SA in an independent cohort from another institution. However, there are still some controversies regarding the quantitative changes in SA occurring in patients with urothelial tumors. In a study performed by Habibi et al. 32, lipid-bound sialic acid (LBSA) and protein-bound sialic acid (PBSA) levels were significantly higher in bladder cancer patients than in normal controls and were significantly correlated with tumor grade, which was in accordance with some earlier reports 10, 33. In contrast, in a report by Lagana et al. 34, the levels of total sialic acid (TSA) and LBSA in patients with bladder tumors were not significantly different from those in normal subjects, which was in agreement with our finding that SA levels in UC patients did not differ significantly from those in patients with papilloma and PUNLMP. In addition, some early studies observed that SA levels were also elevated in benign diseases, such as severe infection 35, type II diabetes 36, rheumatoid arthritis 37 and prostatitis 10, and may serve as a nonspecific acute phase reactant, which may have given rise to bias and disturbed the results of our study. According to the results of our study, we speculate that serum SA levels have strong limitations in the screening of early UC but that they may be useful primarily in the evaluation of the malignant degree of advanced disease and in the localization of urothelial tumors.

However, there were several shortcomings and limitations in this study. First, because the design was analytical and retrospective, the study may have suffered from confounding bias. Besides, the postoperative pathology results collected in clinical practice couldn't provide sufficient information for clinicopathologic stage. Additionally, the gathered clinical information didn't involve patient prognosis, such as relapse-free and overall survival, which would be the focus of our future work.

In conclusion, we believe that SA can be a useful biomarker for the evaluation of the malignant degree of UC and the localization of urothelial tumors. However, further studies are needed to determine whether serum SA levels have applicable clinical value as a single biomarker or as an auxiliary test to traditional approaches in the detection and localization of urothelial tumors.

## Figures and Tables

**Table 1:** Correlations between preoperative SA levels and clinicopathological parameters

Variables	BUC			UTUC		
	No. of patients (%)	SA levels (mg/dL, Mean ± SD)	*p* value	No. of patients (%)	SA levels (mg/dL, Mean ± SD)	*p* value
**Patients**	406	56.41 ± 9.85		185	61.40 ± 12.93	
**Age^a^**						0.743#
≤ 65y	217	55.85 ± 9.46	0.229#	87	61.93 ± 14.63	
> 65y	189	57.06 ± 10.26		98	60.92 ± 11.27	
**Sex**						**0.008#**
Male	345	56.16 ± 9.79	0.176#	103	59.45 ± 13.03	
Female	61	57.85 ± 10.12		82	63.84 ± 12.47	
**Smoking history**						0.188#
Ever	159	56.22 ±9.35	0.931#	58	60.35 ± 14.38	
Never	247	56.54 ± 10.17		127	61.88 ± 12.25	
**Painless macroscopic hematuria**						
Yes	299	56.66 ±9.99	0.436#	125	61.04 ± 12.79	0.647#
No	107	55.72 ± 9.46		60	62.14 ± 13.30	
**WBC^a^**						**0.000#**
≤ 6.16 ×10^9^/L	211	54.01 ± 8.53	**0.000#**	86	57.34 ± 10.07	
> 6.16 ×10^9^/L	195	59.01 ± 10.52		99	64.93 ± 14.11	
**PLT^a^**						**0.000#**
≤ 226 × 109/L	205	53.44 ± 8.09	**0.000#**	97	56.67 ± 10.19	
> 26 × 109/L	201	59.44 ±10.55		88	66.61 ± 13.67	
**AKP^a^**						**0.000#**
≤ 68 U/L	228	53.93 ± 7.41	0.000#	85	56.30 ± 9.01	
> 68 U/L	178	59.59 ± 11.54		100	65.73 ± 14.17	
**LDH^a^**						**0.000#**
≤ 187.50 U/L	207	54.81 ± 8.43	**0.001#**	89	58.49 ± 11.92	
> 187.50 U/L	199	58.08 ± 10.90		96	64.10 ± 13.31	
**PFL^a^**						**0.000#**
≤ 3.09 g/L	229	52.22 ± 5.86	**0.000#**	68	53.49± 8.59	
> 3.09 g/L	177	61.84 ± 11.23		117	65.99 ± 12.84	
**Tumor number**						0.582#
Single	228	55.95 ± 9.94	0.401#	156	61.22 ±12.65	
Multiple	159	56.33 ± 9.52		10	68.02 ± 20.14	
Missing information	19	--		19	--	
**Tumor size^ab^**						**0.001#**
≤ 2.7 cm	226	54.30 ± 7.94	**0.000#**	70	57.43 ± 9.45	
> 2.7 cm	180	59.06 ± 11.29		115	63.81 ± 14.16	
**Pathological characteristics**						0.647*
Papilloma	8	57.40 ± 8.62	0.757*	6	59.26 ± 12.94	
PUNLMP	56	56.01 ± 8.27		4	57.40 ± 14.23	
UC	342	56.46 ± 10.13		175	61.56 ± 12.96	
**Grade**						0.068#
High-grade	194	57.69 ± 11.06	**0.038#**	151	62.40 ± 13.32	
Low-grade	148	54.84 ± 8.52		24	56.32 ± 8.98	
**Tumor invasion**						0.152#
MIUC	169	58.40 ± 11.11	**0.017#**	148	62.21 ± 13.54	
NMIUC	98	55.07 ± 9.31		13	56.61 ± 7.94	
Unknown	75	--		14	--	

Continuous variables are expressed as medians^a^. Bold values are statistically significant (*p* < 0.05). Abbreviations: PUNLMP papillary urothelial neoplasm of low malignant potential; UC urothelial cancer; MIUC muscle-invasive urothelial cancer; NMIUC non-muscle-invasive urothelial cancer. p*: Kruskal-Wallis H-test; *p*^#^: Mann-Whitney U-test.

**Table 2:** The level of serum SA in patients with urothelial tumors

Tumor location	No. of patients (%)	SA levels (mg/dL, Mean ± SD)	*p* value
Bladder	406	56.41 ± 9.85	<0.001*
Ureter	99	59.81 ± 10.37	
Renal pelvis	86	63.23 ± 15.23	

Bold values are statistically significant (*p* < 0.05). p*: Kruskal-Wallis H-test.

**Table 3:** Univariate analysis of preoperative variables prognostic for UC, high-grade UC, MIUC and UTUC

Variables	UC vs. Papilloma & PUNLMP		High grade vs. Low grade		MIUC vs. NMIUC		UTUC vs. BUC	
	HR (95%CI)	*p*	HR (95%CI)	*p*	HR (95%CI)	*p*	HR (95%CI)	*p*
**Age**							1.293 (0.913~1.832)	0.148
*BUC*	1.681 (0.967~2.923)	0.065	1.260 (0.820~1.935)	0.291	1.036 (0.630~1.704)	0.889		
*UTUC*	1.741 (0.475~6.385)	0.403	1.021 (0.430~2.424)	0.962	1.019 (0.327~3.177)	0.974		
**Sex**							0.222 (0.149~0.331)	**0.000**
*BUC*	1.970 (1.022~3.798)	**0.043**	1.354 (0.713~2.571)	0.354	1.129 (0.535~2.383)	0.750		
*UTUC*	0.321 (0.080~1.284)	0.108	3.284 (1.166~9.252)	**0.024**	2.757 (0.729~10.427)	0.135		
**WBC**							1.246 (0.879~1.765)	0.217
*BUC*	1.183 (0.693~2.018)	0.538	1.217 (0.792~1.870)	0.370	1.258 (0.764~2.070)	0.368		
*UTUC*	1.323 (0.361~4.851)	0.673	1.276 (0.533~3.052)	0.584	1.019 (0.327~3.177)	0.974		
**PLT**							0.925 (0.653~1.310)	0.662
*BUC*	1.024 (0.600~1.746)	0.932	1.548 (1.007~2.382)	**0.047**	1.280 (0.777~2.107)	0.333		
*UTUC*	0.369 (0.092~1.475)	0.159	1.188 (0.502~2.814)	0.695	2.250 (0.664~7.629)	0.193		
**AKP**							1.507 (1.063~2.137)	**0.021**
*BUC*	0.732 (0.423~1.268)	0.266	1.251 (0.813~1.926)	0.309	1.016 (0.617~1.673)	0.950		
*UTUC*	1.187 (0.332~4.249)	0.792	1.221 (0.515~2.890)	0.650	1.934 (0.604~6.191)	0.266		
**LDH**							1.122 (0.792~1.589)	0.517
*BUC*	1.412 (0.825~2.417)	0.208	1.660 (1.077~2.559)	**0.022**	1.417 (0.860~2.335)	0.172		
*UTUC*	1.083 (0.303~3.875)	0.902	1.332 (0.561~3.160)	0.516	7.219 (1.546~33.717)	**0.012**		
**PFL**							2.226 (1.557~3.183)	**0.000**
*BUC*	0.741 (0.428~1.284)	0.285	1.418 (0.920~2.186)	0.114	1.467 (0.887~2.427)	0.135		
*UTUC*	1.156 (0.314~4.251)	0.827	1.283 (0.534~3.084)	0.578	1.219 (0.358~4.150)	0.752		
**Tumor size**							2.063 (1.445~2.944)	**0.000**
*BUC*	3.744 (1.965~7.134)	**0.000**	3.137 (2.005~4.908)	**0.000**	4.226 (2.479~7.205)	**0.000**		
*UTUC*	1.692 (0.472~6.068)	0.419	0.520 (0.195~1.385)	0.191	3.917 (1.151~13.325)	**0.029**		
**SA level**				n.d.		n.d.		n.d.
*BUC* (>52.27 * vs ≤52.27)	1.491 (0.828~2.683)	0.183						
*UTUC* (>49.40* vs ≤49.40)	4.194 (1.102~15.962)	**0.035**						
**SA level**		n.d.						n.d.
*BUC* (>61.60 * vs ≤61.60)			2.597 (1.477~4.567)	**0.001**		n.d.		
*UTUC* (>48.35 * vs ≤48.35)			4.770 (1.653~13.760)	**0.004**				
**SA level**								n.d.
*BUC* (>54.35 * vs ≤54.35)		n.d.		n.d.	2.030 (1.225~3.364)	**0.006**		
*UTUC* (>58.35* vs ≤58.35)					3.209 (0.945~10.896)	0.062		
SA level (>60.31* vs ≤60.31)		n.d.		n.d.		n.d.	3.035 (2.099~4.388)	**0.000**

Bold values are statistically significant (*p* < 0.05). Abbreviations: PUNLMP papillary urothelial neoplasm of low malignant potential; UC urothelial cancer; UTUC upper tract urothelial carcinoma; BUC bladder urothelial carcinoma; MIUC muscle-invasive urothelial cancer; NMIUC non-muscle-invasive urothelial cancer; HR hazard ratio; 95% CI 95% confidence interval.

**Table 4:** Multivariate analysis of preoperative variables prognostic for high-grade UC, MIUC and UTUC

Variables	UC vs. Papilloma & PUNLMP		High grade vs. Low grade		MIUC vs. NMIUC		UTUC vs. BUC	
	HR (95%CI)	*p*	HR (95%CI)	*p*	HR (95%CI)	*p*	HR (95%CI)	*p*
**Sex**						0.873	4.128 (2.726~6.250)	**0.000**
*BUC*	1.908 (0.961~3.788)	0.065	1.365 (0.692~2.693)	0.369	0.937 (0.419~2.092)			
*UTUC*	0.194 (0.041~0.923)	**0.039**	2.412 (0.800~7.270)	0.118	3.391 (0.805~14.297)	0.096		
**LDH**							1.080 (0.733~1.591)	0.697
*BUC*	1.521 (0.868~2.667)	0.143	1.595 (1.011~2.518)	**0.045**	1.272 (0.738~2.191)	0.386		
*UTUC*	1.107 (0.283~4.338)	0.884	1.195 (0.482~2.966)	0.701	6.181 (1.208~31.615)	**0.029**		
**PFL**							1.462 (0.957~2.234)	0.079
*BUC*	1.293 (0.708~2.364)	0.403	1.093 (0.660~1.808)	0.731	1.130 (0.613~2.085)	0.695		
*UTUC*	1.983 (0.342~11.490)	0.445	1.194 (0.426~3.341)	0.736	3.126 (0.708~13.808)	0.133		
**Tumor size**							1.660 (1.115~2.474)	**0.013**
*BUC*	3.924 (2.017~7.634)	**0.000**	2.802 (1.761~4.460)	**0.000**	3.985 (2.302~6.899)	**0.000**		
*UTUC*	1.706 (0.440~6.608)	0.440	2.050 (0.730~5.755)	0.173	4.100 (1.081~15.549)	**0.038**		
**SA level**								n.d.
*BUC* (>52.27 * vs ≤52.27)	1.810 (0.959~3.414)	0.067		n.d.		n.d.		
*UTUC* (>49.40* vs ≤49.40)	10.889 (1.720~68.946)	**0.011**						
**SA level**								n.d.
*BUC* (>61.60 * vs ≤61.60)		n.d.	1.941 (1.017~3.707)	**0.044**		n.d.		
*UTUC* (>48.35 * vs ≤48.35)			3.820 (1.108~13.170)	**0.034**				
**SA level**								n.d.
*BUC* (>54.35 * vs ≤54.35)		n.d.		n.d.	1.643 (0.889~3.038)	0.113		
*UTUC* (>58.35* vs ≤58.35)					2.129 (0.477~9.506)	0.322		
**SA** level (>60.31* vs ≤60.31)		n.d.		n.d.		n.d.	2.047 (1.310~3.198)	**0.002**

Bold values are statistically significant (*p* < 0.05). Abbreviations: PUNLMP papillary urothelial neoplasm of low malignant potential; UC urothelial cancer; UTUC upper tract urothelial carcinoma; BUC bladder urothelial carcinoma; MIUC muscle-invasive urothelial cancer; NMIUC non-muscle-invasive urothelial cancer; HR hazard ratio; 95% CI 95% confidence interval.

**Table 5:** The level of serum SA in patients with urothelial tumors in the validation set

Variables	BUC			UTUC		
	No. of patients (%)	SA levels (mg/dL, Mean ± SD)	p value	No. of patients(%)	SA levels (mg/dL, Mean ± SD)	*p* value
Patients	185	60.72 ± 12.72		137	62.98 ± 11.35	
**Grade**			0.062#			**0.002#**
High-grade	138	61.70 ± 13.27		115	64.14 ± 11.13	
Low-grade	47	57.83 ± 10.56		22	56.91 ± 10.77	
**Tumor invasion**			**<0.001#**			**<0.001#**
MIUC	118	63.40 ± 13.67		119	64.53 ± 10.97	
NMIUC	67	56.00 ± 9.18		18	52.72 ± 8.20	

Bold values are statistically significant (*p* < 0.05). Abbreviations: MIUC muscle-invasive urothelial cancer; NMIUC non-muscle-invasive urothelial cancer. *p*^#^: Mann-Whitney U-test.

**Table 6:** The level of serum SA in patients with urothelial tumors in the validation set

Tumor location	No. of patients (%)	SA levels (mg/dL, Mean ± SD)	*p* value
Bladder	185	60.72 ± 12.72	0.041*
Ureter	77	63.75 ± 12.46	
Renal pelvis	60	61.99 ± 9.76	

Bold values are statistically significant (*p* < 0.05). p*: Kruskal-Wallis H-test.

## Abbreviations

UC: urothelial carcinoma
UTUC: upper tract urothelial carcinoma
SA: sialic acid
LBSA: lipid-bound sialic acid
PBSA: protein-bound sialic acid
TSA: total sialic acid
SD: mean ± standard deviation
WBC: white blood cell
LDH: lactate dehydrogenase
PLT: platelet
AKP: alkaline phosphatase
PFL: plasma fibrinogen
ROC: receiver operative characteristic
AUC: the area under the ROC
HR: hazard ratios
95% CI: 95% confidence interval
